# Supplementary material for: Insect Herbivory on Main Stem Enhances Induced Defense of Primary Tillers in Rice (Oryza sativa L.)
Source: Plants (Basel). 2023 Mar 6;12(5):1199. doi: 10.3390/plants12051199 (PMC10005496; doi:10.3390/plants12051199)
Supplement: Supplementary file 1 [file plants-12-01199-s001.zip › plants-2217759-supplementary.pdf]

Supplemental Table S1

TABLE S1. Gene-specific primers for quantitative RT-PCR.

Genes	**Accession NO.**	**Specific Primer**	**Size**
OsAOS	AK068620	F: 5’-CGAGCTCTTCCTCCGATACG-3'	100 bp
		R: 5'-GTCAGAAGGTGGCCTTCTTGAG-3'	
OsAOC	AJ493664	F: 5’-CTGCCTCAACAACTTCACCA-3'	238 bp
		R: 5’-GGATCAGTTCATCGGCTCAT-3'	
OsCOI1	AY168645	F: 5’- TTGCCGTGAATTGGAGTACATAG-3'	115 bp
		R: 5'-GTCAAGTAGCACAAGCCGAAAG-3'	
OsLOX	AF464895	F: 5’-CCGAGCTTGACGCGAAGA -3'	
		R: 5’-GATCGTCGTCGTCCACATTGT -3'	
OsBBPI	HC731644	F: 5’- GCTCATCTGCGAGGACATCT -3'	214 bp
		R: 5’- TTCCTCATGGTCCACACAAG -3'	
OsACTIN	X15865	F: 5’-CTGACGGAGCGTGGTTAC-3'	219 bp
		R: 5’-GGAAGGCGGGAAGAGGAC-3'	
